# The Negative Impact of Insulin Resistance/Hyperinsulinemia on Chronic Heart Failure and the Potential Benefits of Its Screening and Treatment

**DOI:** 10.3390/biomedicines11112928

**Published:** 2023-10-30

**Authors:** Serafino Fazio, Valentina Mercurio, Flora Affuso, Paolo Bellavite

**Affiliations:** 1 Department of Internal Medicine, University of Naples Federico II, 80138 Naples, Italy; 2 Department of Translational Medical Sciences, University of Naples Federico II, 80131 Naples, Italy; valemercurio@yahoo.com; 3 Independent Researcher, 73014 Lecce, Italy; floraaffuso@libero.it; 4 Homeopathic Medical School of Verona, 37121 Verona, Italy

**Keywords:** diabetes, heart failure, insulin resistance, hyperinsulinemia, SGLT2 inhibitors, metformin, berberine

## Abstract

This opinion article highlights the potential alterations caused by insulin resistance and hyperinsulinemia on the cardiovascular system and their negative impact on heart failure (HF), and describes the potential benefits of an early screening with consequent prompt treatment. HF is the final event of several different cardiovascular diseases. Its incidence has been increasing over the last decades because of increased survival from ischemic heart disease thanks to improvements in its treatment (including myocardial revascularization interventions) and the increase in life span. In particular, incidence of HF with preserved ejection fraction (HFpEF) is significantly increasing, and patients with HFpEF often are also affected by diabetes mellitus and insulin resistance (IR), with a prevalence > 45%. Concentric left ventricular (LV) remodeling and diastolic dysfunction are the main structural abnormalities that characterize HFpEF. It is well documented in the literature that IR with chronic hyperinsulinemia, besides causing type 2 diabetes mellitus, can cause numerous cardiovascular alterations, including endothelial dysfunction and increased wall thicknesses of the left ventricle with concentric remodeling and diastolic dysfunction. Therefore, it is conceivable that IR might play a major role in the pathophysiology and the progressive worsening of HF. To date, several substances have been shown to reduce IR/hyperinsulinemia and have beneficial clinical effects in patients with HF, including SGLT2 inhibitors, metformin, and berberine. For this reason, an early screening of IR could be advisable in subjects at risk and in patients with heart failure, to promptly intervene with appropriate therapy. Future studies aimed at comparing the efficacy of the substances used both alone and in association are needed.

## 1. Introduction

Chronic heart failure (HF) represents the final clinical evolution common to several cardiovascular diseases characterized by different specific etiology and pathophysiology. HF is the leading cause of hospitalizations in individuals aged >65 years. The crude age-adjusted incidence ranges from 1 to 5 cases per thousand person-years, and there is an exponential increase in incidence with advancing age. Its prevalence ranges from 3 to 20 individuals per 1000 people over 65 years of age.

Chronic HF is still burdened by high mortality: only 35% of patients are alive 5 years after the first diagnosis of HF. The mortality of subjects with HF is 6–7 times higher than the general population in the same age group [1]. Insulin resistance (IR)/hyperinsulinemia (Hyperins) is a pathogenic factor of type 2 diabetes, associated with metabolic syndrome, hypertension, low HDL cholesterol, and hypertriglyceridemia, but may also be present in a significant proportion of healthy subjects of normal weight. It affects about 40% of the population in Western countries and is associated with incident symptomatic cardiovascular disease [2].

Chronic HF has been classified into three different phenotypes based on the values of the measured left ventricular ejection fraction (LVEF):

I. Heart failure with reduced ejection fraction (HFrEF) is defined by an LVEF ≤ 40%.
II. Heart failure with mildly reduced EF (HFmrEF) is defined by an EF between 41% and 49%.
III. Heart failure with preserved EF (HFpEF) is defined by an LVEF ≥ 50% in the presence of symptoms and signs of HF associated with structural and/or functional cardiac abnormalities and/or increased natriuretic peptides [1].

HFpEF accounts for about 45% of all HF cases and has been on the rise in the recent years [3]. In fact, due to the increased scientific interest in HF and improved characterization and modern diagnostic tools, the incidence of HFrEF tends to decrease in percentage, while HFpEF tends to increase [4].

Increased left ventricular mass (LVM) is a stronger predictor of HF than coronary artery calcium score [5]. Increased LVM, as measured by echocardiogram, was also found to be an independent risk factor of a depressed EF [6]. The majority of patients with HFpEF show a concentric pattern of LV remodeling, characterized mainly by: A. Normal or near normal LV end-diastolic volume; B. Increased parietal thickness and/or LVM; C. Increased ratio of LVM to cavity volume; D. Increased relative wall thickness (RWT) defined as 2× posterior wall thickness (PWT)/LV end-diastolic diameter (LVDD), or as septal WT+PWT/LVDD [3]. The most common cause of HFpEF is diastolic LV dysfunction, secondary to increased stiffness of a hypertrophied LV causing elevation of LV filling pressures.

Data from clinical studies show that diabetes mellitus (DM) has a prevalence of about 45% in individuals with HFpEF, but IR certainly has a higher prevalence [7]. Generalized IR is strictly associated with HF, and this increases the risk of hospitalization, cardiovascular deaths, and all-cause deaths. The changes in insulin signaling, due to IR, within cardiomyocytes and vascular smooth muscle cells alter cardiac function, thereby worsening HF [8]. 

In this opinion article, we describe the potential alterations caused by IR/Hyperins on the cardiovascular system, highlighting its negative impact in HF, and discuss the possibility of early screening and treatment of IR to improve prognosis in HF patients. In this regard, we reviewed the scientific literature, selecting articles on PubMed, Scopus, and Science Direct using the following key terms: heart failure, cardiovascular system, insulin resistance, hyperinsulinemia, diabetes mellitus, treatment of insulin resistance, SGLT2 Inhibitors, metformin, berberine, GLP-1 receptor agonists. We collected and analyzed laboratory and clinical research studies describing the effects of IR/Hyperins on the cardiovascular system, and more specifically in HF. Furthermore, we analyzed the manuscripts describing the potential beneficial effects of reducing IR/Hyperins through treatment with some known substances.

## 2. Insulin Resistance/Hyperinsulinemia and Chronic Heart Failure

IR is a pathological condition characterized by a decrease in sensitivity and response to the metabolic actions of insulin. In fact, at a given concentration of this hormone, the biological effects are clearly less than expected. Increased insulin levels (hyperinsulinemia) are necessary in order to maintain normal glucose tolerance and, therefore, hyperinsulinemia is a defining event of IR [9,10].

IR turns out to be a key mechanism in the development of type 2 diabetes, systemic hypertension, HF, and other various cardiovascular diseases. IR is the earliest abnormality in the natural history of type 2 diabetes, due to defects of insulin receptor function, and the chronic insulin over-secretion results in progressive pancreatic beta cell dysfunction [9,10]. Some studies have shown that hyperinsulinemia, resulting from IR, anticipates the development of overt type 2 diabetes by as much as 10 to 15 years. In the majority of cases, IR is asymptomatic or paucisymptomatic, and affected subjects, when identified, are mostly referred for lifestyle modifications with frequent non-adherence to such indications [9,10]. While it can be easily suspected in the context of metabolic syndrome and polycystic ovary syndrome, the diagnosis of IR/Hyperins becomes difficult in the case of normal-weight or thin subjects. The diagnosis, however, should be done as early as possible to initiate prompt therapeutic interventions.

The diagnostic gold standard for IR is the hyperinsulinemic euglycemic clamp, but this test is poorly applicable for screening purposes, because of its difficulty, time of performance and cost. For clinical screening, several easier-to-obtain substitute indices have been evaluated, such as the Homeostasis Model Assessment of IR index (HOMA-IR) and the Triglyceride–Glucose Index (TyG). HOMA-IR is calculated by multiplying the fasting blood glucose value in mmol/L × the fasting insulinemia value in mU/L and dividing the result by 22.5 [(glycemia × insulinemia)/22.5]. The TyG index is obtained by calculating the natural logarithm of the product between the fasting triglyceride (Tg) value in mg/dl and the fasting blood sugar (FBG) value in mg/dl, then dividing the result by 2 [logn (serum Tg × FBG)/2]. Both indices showed good correlation with clamp data. A HOMA-IR index between 0.23 and 2.5 can be considered normal in the adult population, while the cut-off value for the TyG index is 4.5 [11,12].

Chronic long-lasting hyperinsulinemia, typical of IR, produces damage in target organs, and one of the recognized targets of insulin is certainly the cardiovascular system [13]. Indeed, insulin exerts various pathophysiological actions on the heart and vessels, since insulin receptors are highly expressed in the myocardiocytes and vascular smooth muscle cells. Insulin signaling regulates heart growth, survival, substrate uptake and utilization, and mitochondrial metabolism. Consequently, impaired insulin signaling may contribute to the development of pathological ventricular remodeling and progression of HF [14].

Insulin is also a growth factor, and both insulin and insulin like growth factor-1 (IGF-1) are able to bind to and activate each other’s receptors, even if with lower affinity. Both the insulin receptor (InsR) and the IGF-1 receptor (IGF-1 R) elicit a common downstream signaling resulting in the activation, by phosphorylation of a tyrosine kinase, of two important pathways: that of phosphoinositide-3 kinase (PI3K/Akt), and that of Shc-Ras-mitogen activated protein kinase (MAPK) [15]. The PI3K/Akt pathway is an intracellular signal transduction pathway that promotes important actions in response to extracellular signals, but predominantly mediates the metabolic actions of insulin, regulating glucose metabolism in muscle and in adipose and hepatic tissues; it also regulates nitric oxide (NO) formation by vascular endothelial and smooth muscle cells. The MAPKs are among the most ancient signal transduction pathways which coordinately regulate gene expression, mitosis, survival, apoptosis, differentiation, etc. and primarily mediate mitogenic and proliferative actions of insulin; furthermore, they stimulate endothelial cells to form increased amounts of the vasoconstrictor endothelin-1(ET-1) and increase the expression of adhesion molecules on the vascular endothelium [16,17]. Under normal conditions, these two pathways are in balance and help to maintain vascular homeostasis: the first pathway, stimulating NO production, causes vasodilatation and reduction in vascular resistance with increased blood flow to tissues, while the second one, stimulating ET-1 formation, causes vasoconstriction and activates the sympathetic system, leading to a hypertensive pattern and accelerating the development of cardiac hypertrophy and atherosclerosis.

**Figure 1:**
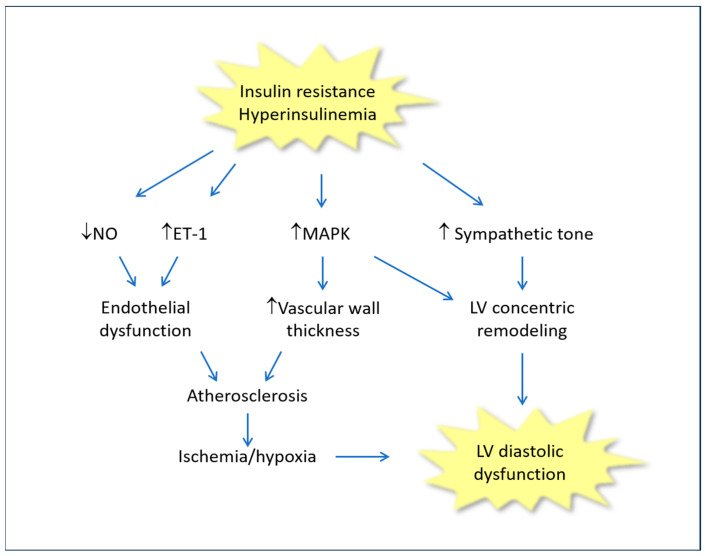
Molecular mechanisms involved in the cardiac consequences of insulin resistance and hyperinsulinemia. ET-1: Endothelin-1; LV: Left ventricle; MAPK: Mitogen-activated protein kinase; NO: Nitric oxide.

The main mechanisms by which IR/Hyperins lead to left ventricular failure are summarized in Figure 1.

Under conditions of IR, the PI3K-dependent pathway is specifically altered while the MAPK-dependent pathway is altered little or not at all. This is why the hyperinsulinemia that is typically associated with IR, in an attempt to maintain glucose levels in the normal range, ends up increasing the activity of the MAPK pathway, with prevalence of deleterious effects at the cardiovascular level due to mitogenic and proliferative actions, and to over exposure to ET-1, which, in the presence of reduced NO production, results in serious endothelial dysfunction [18]. Chronic hyperinsulinemia can result in arterial hypertension not only for the elevated levels of ET-1 and the increased sympathetic tone, but also for a documented anti natriuretic effect of insulin [19]. In addition, chronically increased insulin levels may produce mitogenic and proliferative effects on the cardiovascular system, as they result in proliferation of vascular smooth muscle cells and increased LVM with concentric remodeling [20]. As mentioned above, the cardiovascular changes produced by hyperinsulinemia may act misrecognized for years in the presence of IR until overt type 2 diabetes appears. For this reason, most individuals who develop overt type 2 diabetes after years of IR, already show evident cardiovascular abnormalities at first diagnosis, such that, according to guidelines, they are subject to a higher degree of severity in cardiovascular prevention and treatment lines [21].

Alzadjali et al. (2009) [22] demonstrated how IR is highly prevalent among non-diabetic HF patients (67%). In such patients, the fasting IR index increased progressively with worsening New York Heart Association classes. In addition, patients with HF and IR demonstrated significantly reduced exercise capacity and peak O_2_ achieved, compared with patients with HF alone. Unfortunately, no distinction was made between patients with HFrEF and patients with HFpEF in this study [22]. There is a large scientific literature highlighting that IR/Hyperins is associated with pathological remodeling of the LV. It is well known that LVH is a constant feature of diabetic cardiomyopathy [23]. In fact, LVH is very prevalent in patients with type 2 diabetes and is a strong predictor of cardiovascular adverse events, in particular of HFpEF. LVH can result in impediment to normal LV filling and can lead to diastolic HF [24]. In these cases, intervention with drugs such as angiotensin-converting enzyme inhibitors, sartans, etc., which result in LVH reduction, also improves the prognosis of this condition [25]. However, the causes of LVH in subjects with diabetes are not fully elucidated. Some researchers have hypothesized and demonstrated that increased fat deposition in the LV myocardium may also contribute to the increased ventricular mass, and that excess lipid in the myocardiocytes may alter cell signaling and cardiac structure by accumulation of toxic lipid species (lipotoxicity), resulting in abnormal heart function [26,27,28]. Other researchers have shown that IR/Hyperins and waist-to-hip ratio are associated with LV concentric remodeling regardless of BMI levels, suggesting that treating IR could give a greater boost to the regression of LV concentric remodeling and improve the prognosis regarding the development of HF [29]. Diabetes is associated with an increased risk of morbidity and mortality in patients with HFrEF, HFmrEF, and, especially, in patients with HFpEF. DM in patients with HFpEF is associated with a 2-fold increase in the risk of cardiovascular death and HF-related hospitalizations, and with an increased risk of all-cause mortality [30,31]. In the digoxin study, DM was found to be associated with a 68% increased risk of HF hospitalization and death [32]. This was later confirmed in the I-Preserve study [33]. In the RELAX study, it was shown that subjects with HFpEF and DM had similar levels of NT-ProBNP compared with nondiabetics, but markedly higher levels of the vasoconstrictor endothelin-1 (ET-1), of markers of inflammation such as uric acid and C-reactive protein, and of profibrotic markers such as valectin-3 and carboxy-terminal telopeptide of collagen type 1 [34]. It is well known that the alterations of these parameters are also characteristic of IR/Hyperin states [35]. Echocardiographically, patients with HF and DM had a significantly abnormal LV diastolic function as shown by higher E/e’ ratio, with increased LVM and reduced exercise tolerance compared with subjects with HF without DM [36].

Recently, it has been reported that, due to the advancement in HF treatment, a relevant number of patients with HFrEF and HFmrEF have experienced an improvement of LVEF, such that it is almost back to normal value. This condition has been termed HF with recovered EF (HFrecEF). We have seen how IR is highly present in the subjects with HF and is closely related to prognosis. Patients with HF and IR had significantly greater difficulty in improving their LVEF, and so the authors conclude that IR is independently associated with a failure to improve LVEF in subjects with HFrEF [37]. It is well known that patients with type 2 DM have longstanding IR/Hyperins before they reach the stage of overt DM, and this contributes to much of the pathologic cardiovascular changes already present in the diabetic subjects at first diagnosis.

## 3. Treatment of Insulin Resistance/Hyperinsulinemia: Possible Beneficial Effects in Patients with Chronic HF

The metabolic and organic imbalances described are very complex and there are many different therapeutic approaches that may be considered for the treatment of IR/Hyperins.

### 3.1. Role of Diet

The gold standard for the treatment of IR/Hyperins is certainly constituted by following a balanced diet, low in carbohydrates, low in calories in obese and overweight subjects, and physical activity, when possible, moderate and constant [38]. Among the various diets, very low-calorie ketogenic diets (VLCKDs), which have been used with good results in obese or overweight subjects with prediabetes or diabetes, deserve special mention. VLCKDs are calorie-restricted diets (<800 Kcal/day but >600 Kcal/day) with a low carbohydrate content (between 20 and 60 g/day). According to international guidelines, VLCKDs can be used continuously for 12 weeks, always under medical supervision [39].

When the intake of carbohydrates is drastically reduced, the consequent modification of the relationship between the concentration of insulin and that of glucagon promotes the mobilization of lipids from tissue deposits and the oxidation for energy purposes.

In practice, the drastic reduction in the intake of carbohydrates determines an increase in fat catabolism, with an imbalance between the production of pyruvate, oxaloacetate and acetyl-Coenzyme A (acetyl-CoA). Excess acetyl-CoA is transformed in the liver cells into the three ketone bodies: acetoacetate, hydroxybutyrate, and acetone. Myocardium, skeletal muscle, and brain capture and can use the first two. At the pancreatic level, these contribute to metabolic improvement in patients with IR.

Normally, the heart’s large demand for energy is met mainly by the mitochondrial oxidation of fatty acids and glucose. In HF, there is a reduction in mitochondrial oxidative metabolism and glucose oxidation, which makes the heart starved of energy. Ketone bodies can be rapidly oxidized by the heart muscle and can provide a replacement and additional energy source to the failing heart. This mechanism may become an adaptive response of the heart to reduce the severity of HF [40].

However, as is well known from clinical practice, maintaining a correct lifestyle is consistently performed only by a low percentage of patients, while the majority, after a variable duration, return to a detrimental lifestyle. To improve adherence to these indications, patients must be well informed, and reminded often, of the risks they incur by not adhering to these indications. Furthermore, we are also convinced that, to reduce hospital admissions of patients for HF exacerbation, they must be followed consistently at home via telemedicine or through a periodic monthly visit by a nurse specialized in caring for patients with HF [41].

### 3.2. Sodium-Glucose Cotransporter-2 Inhibitors

Sodium-glucose cotransporter-2 (SGLT2) inhibitors are a class of oral drugs approved by the US FDA for use with diet and exercise to lower blood sugar in adult patients with type2 diabetes and recently included in the guidelines for treatment of HF [1]. They include canaglifozin, dapaglifozin, and empaglifozin. It has been shown that treatment with SGLT2 inhibitors reduces the risk of hospitalization, death from cardiovascular events, and all-cause mortality in patients with HF [42]. In a recent study, 6263 patients > 40 years of age with an EF > 40%, assigned to receive dapaglifozin (10 mg once a day) or placebo in addition to standard therapy, showed a significant reduction in the combined risk of worsening HF and death from cardiovascular events. This result was similar in both patients with EF ≥ 60% and those with EF < 60% [43]. This beneficial effect of SGLT2 inhibitors in patients with HFpEF has also been confirmed by a recent meta-analysis study performed on 12,251 patients drawn from the DELIVER and EMPEROR-Preserved studies. Indeed, it shows that treatment with SGLT2 inhibitors results in a consistent reduction in both deaths from cardiovascular events and first hospitalization for HF [44]. The exact mechanisms by which these drugs beneficially act in HF remain elusive. However, the suggested mechanisms are: blood volume regulation, cardiorenal mechanisms, metabolic effects, improved cardiac remodeling, direct effects on contractility and sodium ion homeostasis, reduction of inflammation and oxidative stress, etc. [45]. Administration of an SGLT2 inhibitor to subjects with IR/Hyperins reduces the amount of glucose disposal required to maintain blood glucose in the normal range as result of increased urinary glucose excretion. Therefore, the amount of insulin required for a given amount of glucose disposal will be reduced. As a consequence, average circulating insulin levels will be lower than before treatment [46]. SGLT2 inhibitors reduce IR and circulating insulin levels, thereby reducing their adverse cardiovascular effects. Cardiac remodeling is a constant component of IR/Hyperins and an important determinant of the development and progression of HF. Counteracting pathological cardiac remodeling is likely to be a central mechanism in determining the cardioprotective benefits of SGLT2 inhibitors.

Treatment with SGLT2 inhibitors has been shown to favorably affect cardiac remodeling through myocardial, mitochondrial, interstitial, vascular, autonomic nervous system, etc. applications [47]. Results of a meta-analysis study of randomized and controlled trials, comparing the effects of SGLT2 inhibitor treatment versus placebo on changes in LVM, assessed by cardiac magnetic resonance imaging, showed that such therapy significantly reduced LVM [48]. In particular, the reduction in LVM and concentric remodeling produce an improvement of diastolic function and an increase in EF. Very recently, some researchers have reported that a treatment of six months with dapaglifozin in 27 patients with type 2 DM significantly reduced the epicardial adipose tissue thickness (evaluated by echocardiography), HbA_1_c, body weight, BMI, and blood glucose, while the LV global longitudinal strain (evaluated as an index of LV systolic function by m-mode echocardiography) was increased. These data further support the beneficial effects of SGLT2 inhibitors on the heart, although all of these effects have to be confirmed in the long term [49]. In addition, 12 large-scale trials involving more than 70,000 patients with HF showed that long-term SGLT2 inhibition produced a relevant 20 to 25% decrease in the combined risk of cardiovascular deaths or hospitalizations for HF, providing insights unto the durability and sustainability of their effects [50]. Due to their beneficial effects on the outcomes of patients with HF, SGLT2 inhibitors have entered the guidelines for the treatment of HF. However, further studies are needed to monitor any long-term adverse effects.

### 3.3. Metformin

Metformin, an oral antidiabetic belonging to the biguanide class and used for many years in the treatment of type 2 diabetes mellitus, has been amply demonstrated to reduce IR/Hyperins and has shown beneficial effects in patients with HFpEF. In fact, the results of a review and meta-regression analysis study have shown that treatment with metformin reduces mortality in patients with HF, although larger positive effects (*p* < 0.003) were demonstrated in the subgroup of patients with HFpEF [51]. Indeed, metformin has been shown to have numerous favorable effects in HF patients, such as, for example, the improvement of myocardial energy status consequent to modulation of glucose and lipid metabolism, the reduction in oxidative stress and inflammation, and the reduction in cardiac pathologic remodeling [52]. Oxidative stress and inflammation are mechanisms and significant drivers in the development and progression of cardiovascular diseases. Experimental and clinical studies have demonstrated that oxidative stress and inflammation associated with conditions of IR, such as obesity, hypertension, and diabetes produce LV hypertrophy, fibrosis, diastolic dysfunction, HF, and ischemia/reperfusion damage [53]. Therefore, it seems obvious that determining a reduction in these parameters can produce a series of beneficial effects at the cardiovascular level. A recent meta-analysis has shown that metformin treatment results in favorable effects on LVM and LVEF, both in patients with and those without pre-existing cardiovascular disease [54]. Few years ago, another interesting study, double-blind and placebo-controlled, showed that, in non-diabetic but insulin resistant patients with HF, metformin treatment for 4 months produced a significant (*p* < 0.001) improvement in IR indices, accompanied by a significant (*p* < 0.036) reduction in the minute ventilation/carbon dioxide production (VE/VCO_2_) slope [55]. The VE/VCO_2_ slope has a strong predictive value in patients with HF, and the risk of mortality is thought to increase significantly when the value is >32.8 [56].

### 3.4. Berberine

Many natural substances have shown beneficial effects on IR/Hyperins. Some studies demonstrated the beneficial effects of berberine, quercetin, and silymarin on IR/Hyperins and the cardiovascular system. From the analysis of experimental in vitro and in vivo studies, it clearly appears that these three substances reduce IR and consequent circulating elevated insulin levels. Therefore, these substances could protect the evolution toward overt type 2 diabetes and the development of cardiovascular abnormalities [35]. Both quercetin and silymarin have extensive literature that support their efficacy in counteracting IR/Hyperins and LVH development, and in preventing cardiovascular alterations [57,58,59,60,61,62,63,64,65]. However, these experimental studies have not been followed by clinical studies to confirm their beneficial effects in humans. Berberine, in contrast, has many clinical studies supporting its efficacy in counteracting IR/Hyperins and, consequently, in protecting against the development of IR-related cardiovascular damage.

Berberine is a vegetable alkaloid in use for over 2000 years in Chinese and Ayurvedic medicines, which has been shown to have numerous beneficial effects on human health. It is contained in numerous plants, including *Hydrastis canadensis* and *Coptis chinensis*. A study designed to assess the efficacy of berberine to produce improvements in patients with HF, was carried out on patients with HF divided into two groups: a group of 79 patients treated with berberine and a group of 76 patients treated with a placebo in addition to standard therapy, according to guidelines, for HF. Evaluation of the parameters considered was done basally, after 8 weeks and after an average of 24 months of follow-up. Berberine treatment produced a significant increase in EF and exercise capacity and a reduction in the frequency and complexity of ventricular premature beats. In addition, during the 2-year follow-up, 7 patients died in the berberine group and 13 patients in the placebo group (*p* < 0.02) [66].

A further study evaluated the relationship between the clinical effects of berberine at a dose of 1.2 g per day in patients with severe congestive HF and the hematic concentrations of berberine assessed by HPLC. It was shown that berberine decreased the number and complexity of premature ventricular beats and increased EF more in patients whose plasma concentration of berberine was >0.11 mg/L than in those with lower concentrations [67]. In a randomized, double-blind, placebo-controlled trial of 18 weeks, 59 patients with metabolic syndrome were treated with a nutraceutical combination containing 500 mg of berberine or with placebo once daily. The results showed a significant decrease (*p* < 0.05) in HOMA-IR and fasting insulin levels in the berberine-treated group [68]. Also, these patients showed a significant decrease in LVM, RWT, and diastolic dysfunction, evaluated by Doppler-echocardiography, after treatment in comparison with the placebo group [68]. These results were confirmed by a further multicenter, randomized, double-blind, placebo-controlled trial performed in 145 patients with metabolic syndrome and LVH. The patients were divided into two groups; the first group of 74 patients was given a nutraceutical combination containing berberine at 500 mg daily, and the second group (71 patients) were given a placebo. The duration of the trial was six months. The results showed that the treated group had a significant reduction in LVM (*p* < 0.001) as compared with the placebo group. The authors concluded that the treatment could represent an effective strategy to reduce the cardiovascular risk [69]. Unfortunately, despite the beneficial effects demonstrated at the cardiovascular level by these substances both in experimental studies and in some clinical studies, they have so far not been taken into consideration for a trial demonstrating clear clinical efficacy in patients with HF.

### 3.5. Glucagon-like Peptide-1 Receptor Agonists

Glucagon-like peptide-1 is a hormone produced by the intestine that stimulates insulin secretion and inhibits glucagon secretion by the pancreas. Among other things, it slows down gastric emptying, increasing the sense of satiety, and reduces appetite by acting directly on the hunger regulation centers in the central nervous system, thus helping to lose weight [70].

Glucagon-like peptide-1 receptor agonists (GLP-1 RAs) are substances utilized in the treatment of type 2 diabetes and obesity. The ones currently in use are: semaglutide, albiglutide, delaglutide, exenatide, liraglutide and lixisenatide. Despite the positive reports on the beneficial effects of treatment with GLP-1 RAs in the treatment of diabetes and obesity, the data in the scientific literature on the use of GLP-1 RAs in subjects with HF are contrasting. In fact, a recent meta-analysis study of 54,092 participants of 7 randomized controlled trials on the use of GLP-1 RAs in subjects with type 2 diabetes, of whom 16% also had a history of HF, showed that these drugs may help prevent new onset of HF in the diabetic population, while, on the other hand, in subjects with pre-existing HF, they did not reduce the risk of HF exacerbation or mortality [71]. A further recent meta-analysis study, performed to verify whether the use of GLP-1 RAs in patients with HF with or without type 2 diabetes could improve morbidity and mortality compared to placebo treatment, also demonstrated that the GLP-1 RAs did not lead to an improvement in major adverse cardiovascular events, including cardiovascular mortality, or a reduction in hospitalizations for HF. Furthermore, they did not determine an improvement in EF and in the six-minute walking test [72].

### 3.6. Interactions of Some Drugs, Used in HF, with IR/Hyperins

Some older drugs, still quite often used by patients with HF, while improving the overall prognosis of these patients, can induce or worsen a state of IR/Hyperins. The drugs most commonly used are beta-blockers, thiazide diuretics, and statins. The beta-blockers that have a greater negative action on IR/Hyperins are predominantly those of the first generation, not beta 1 selective and without vasodilating action, such as propranolol [73,74]. As far as thiazide diuretics are concerned, the negative action on IR/Hyperins has been abundantly confirmed, particularly at high doses, which are most commonly used in HF states [75,76,77]. Statins are drugs widely used in cardiovascular prevention for the treatment of hypercholesterolemia. Their efficacy on cardiovascular morbidity and mortality has been sufficiently demonstrated, and this justifies their use. However, they can determine or worsen a state of IR/Hyperins, which, as we have seen, can have a negative effect on the cardiovascular system and partially reduce the positive action of statins [78]. Probably, their cardiovascular prevention action would be even more positive if they did not have this negative action towards IR. What has been said should lead us to contrast the potential negative action of these drugs against IR/Hyperins, where possible, with lifestyle changes and with the use of substances that have demonstrated efficacy against IR/Hyperins. This could determine a favorable synergistic action that could add efficacy to their positive action on cardiovascular morbidity and mortality.

## 4. Conclusions

Despite the considerable progress in the prevention and treatment of heart diseases, HF is still a very important cause of recurring hospitalizations, with relevant social and health care costs, and it is burdened with a significant mortality. It is also noteworthy, not only for treatment purposes, to highlight the increase in the incidence of heart failure. Among the causes that could have contributed to this increase could be the increase in the incidence of IR and consequent hyperinsulinemia, and diabetes. It is known that IR is closely related to metabolic syndrome, which is in a phase of progressive diffusion and increase in developed and developing countries. Elevated circulating insulin levels from IR could contribute to the development and progressive worsening of HF. In fact, a considerable prevalence of IR has been demonstrated in HF. Hyperinsulinemia, as amply demonstrated in the scientific literature, can, over the years, cause progressive alterations at the cardiovascular level. For this reason, early screening of IR/Hyperins in at-risk subjects and in patients with HF could certainly be useful, given that we now have numerous substances that have been shown to be able to counteract it and, probably, reduce and slow down the progression toward type 2 diabetes and cardiovascular damage. What the best drug and the correct dosage might be, or whether it is better to use a combination of drugs, is not currently known. For these reasons, dose-response trials and head-to-head comparisons are necessary to help optimize the treatment.

## Data Availability

Data sharing not applicable.
